# Handgrip force steadiness in young and older adults: a reproducibility study

**DOI:** 10.1186/s12891-018-2015-9

**Published:** 2018-04-02

**Authors:** Andreas W. Blomkvist, Fredrik Eika, Eling D. de Bruin, Stig Andersen, Martin Jorgensen

**Affiliations:** 1 Department of Geriatric and Internal Medicine, Aalborg University Hospital, Copenhagen, Denmark; 2 Institute of Human Movement Sciences and Sport, Department Health Sciences and Technology, ETH Zurich, HCP H 25.1, Leopold-Ruzicka-Weg 4, CH-8093 Zürich, Switzerland; 3 Division of Physiotherapy, Department of Neurobiology, Care Sciences and Society, Karolinska Institutet, SE141, 83 Huddinge, Sweden

**Keywords:** Nintendo Wii balance board, Reproducibility, Reliability, Force steadiness, Handgrip steadiness

## Abstract

**Background:**

Force steadiness is a quantitative measure of the ability to control muscle tonus. It is an independent predictor of functional performance and has shown to correlate well with different degrees of motor impairment following stroke. Despite being clinically relevant, few studies have assessed the validity of measuring force steadiness. The aim of this study was to explore the reproducibility of handgrip force steadiness, and to assess age difference in steadiness.

**Method:**

Intrarater reproducibility (the degree to which a rating gives consistent result on separate occasions) was investigated in a test-retest design with seven days between sessions. Ten young and thirty older adults were recruited and handgrip steadiness was tested at 5%, 10% and 25% of maximum voluntary contraction (MVC) using Nintendo Wii Balance Board (WBB). Coefficients of variation were calculated from the mean force produced (CVM) and the target force (CVT). Area between the force curve and the target force line (Area) was also calculated. For the older adults we explored reliability using intraclass correlation coefficient (ICC) and agreement using standard error of measurement (SEM), limits of agreement (LOA) and smallest real difference (SRD).

**Results:**

A systematic improvement in handgrip steadiness was found between sessions for all measures (CVM, CVT, Area). CVM and CVT at 5% of MVC showed good to high reliability, while Area had poor reliability for all percentages of MVC. Averaged ICC for CVM, CVT and Area was 0.815, 0.806 and 0.464, respectively. Averaged ICC on 5%, 10%, and 25% of MVC was 0.751, 0.667 and 0.668, respectively. Measures of agreement showed similar trends with better results for CVM and CVT than for Area. Young adults had better handgrip steadiness than older adults across all measures.

**Conclusion:**

The CVM and CVT measures demonstrated good reproducibility at lower percentages of MVC using the WBB, and could become relevant measures in the clinical setting. The Area measure had poor reproducibility. Young adults have better handgrip steadiness than old adults.

## Background

Diminished strength, especially maximal voluntary contraction (MVC), and greater variability of voluntary contractions develops with advancing age and with neurological insults such as stroke [1]. However, everyday tasks such as walking or holding items require steady and sustained sub-maximal contractions rather than maximal force. Force steadiness is a quantitative measure of this ability and might be a better proxy for functional limitations than MVC. It is most often assessed by measuring the variability of force production as the subject aims to maintain a target force [2]. People with a history of stroke have impaired isometric steadiness compared to controls, and the degree of impairment correlates with functional tests [3] and clinical measures of motor impairment [4–6]. Moreover, the degree of force variability is correlated with the severity of stroke, making it a functionally relevant index of motor performance [5].

Force steadiness has also been considered an independent predictor of functional performance in healthy individuals, e.g. chair-rise time, stair-climbing and postural sway [7, 8]. Furthermore, hand muscle steadiness correlates with different hand performance tasks and, interestingly, more strongly so than maximal strength [9]. Exercise interventions have shown similar improvements in steadiness and functional tasks, making it an index of hand function [10–14]. Although studies on age-related differences in force steadiness have given varying results, older adults seem to have reduced hand muscle steadiness compared to younger individuals, especially at lower forces [15–18]. However, force steadiness depends not only on age, but also on the muscle group tested [19], on the type and intensity of muscle contraction, physical activity level of the individual [20], and the use of different experimental methods and measures.

Most commonly, the force produced during MVC is measured and a percentage of this force used as a target force, e.g. 5%, 20% or 40% of MVC. The participant then tries to match his or her force output with the target force (usually via visual feedback) for a given duration. From this, one can derive the standard deviation (SD) of the force variability and the coefficient of variation (CV). However, other measures are frequently calculated, such as the ApEn^1^ of the time-dependent signal [5], CV using the target force [16] or approximating the area between target force line and force curve produced [21].

So far, only a few studies have investigated the reliability of measures on force steadiness [22–27]. In general, results are mixed, but usually poorer compared to values seen with maximal strength assessments. More importantly, there seem to be no reports on the reliability or agreement of force steadiness measurements on hand muscles.

Credible reliability is a prerequisite for the use of different measures in clinical settings. A related measure is the duration of sustained handgrip contraction at a given target force (e.g. 50% of MVC). Despite being used in previous research [28–30], the reliability of this measure has been found to be poor and not recommendable for clinical use [31]. This emphasises the importance of method validation before using measures of force steadiness in the clinical setting. If valid and feasible, such measures could have value in clinical settings, e.g. as an outcome measure for rehabilitation programs in stroke survivors [5]. Therefore, we aim to test an alternative method and measure of force steadiness using the Nintendo Wii Balance Board (WBB). The WBB has previously shown promising results as a valid instrument for the assessment of balance [32–34], reaction time [35], isometric handgrip strength [36] and whole isometric lower limb strength [37, 38].

The aim of this study was to explore the reproducibility of the WBB to measure handgrip force steadiness in healthy older adults, and to assess the purported difference in force steadiness between young and old adults.

## Method

### Design and terminology

A method comparison study with the gold standard is usually warranted when introducing a new method [39]. We used a test-retest design with seven days between each session to test the reproducibility of the WBB method [39]. The term “reproducibility” refers to the variation in measurements made on a subject under changing conditions [39]. Reproducibility was the ability of our method to give consistent results on two occasions separated in time. This includes the ability to distinguish between subjects in a sample on repeated tests, referred to as reliability [39, 40], and the degree to which two measurements made on the same subject are comparable, referred to as agreement [40]. Following the Guidelines for Reporting Reliability and Agreement Studies, we use the terms reliability and agreement [40].

### Study-population

Our study-population consisted of a group of old adults for reproducibility testing and a smaller group of young adults for age-related comparison. The old participants were recruited by telephone lists in Ålesund municipality in Møre and Romsdal County, Norway. Participants were included if they were 55 years or older, judged themselves to be healthy and willing and able to be tested twice, one week apart. Participants were excluded if they had neuromuscular diseases (e.g. Parkinson’s disease), sequelae from stroke, suffered from dementia, did not understand Norwegian, suffered from acute illness or had recent (within 6 months) surgery.

The younger participants were recruited at university campus at the University of Oslo. The inclusion and exclusion criteria were the same for both groups, but age was between 20 and 30 years for the younger participants. All participants gave oral consent and the protocol was reviewed and declared not mandatory for submission by the Regional Committee for Medical and Health Research Ethics in Norway (2016/1505/REK Nord). The North Regional Ethics Committee, Norway, did not provide a full ethical evaluation and formal approval due to the ethically benign nature of our work (method development study). Still, all participants were required to provide oral consent to participate in the study and, thus, participant registration was synonymous with documented consent. None of the authors had access to any personal or potentially identifying participant information.

### Experimental procedure

The WBB is a rectangular-shaped platform with one force transducer in each corner. Data was wirelessly streamed to a computer (Samsung Chronos series 7, Windows 8) and onto FysioMeter software (version 1.0.8, Bronderslev, Denmark) via a Bluetooth Human Interface Device. Each of the transducers channels delivered 16-bit digital data at approximately 100 Hz. These were filtered using 4th order Butterworth filter (cut-off 20 Hz). The software records and visualizes the data in real-time on a force-time curve.

**Fig. 1:**
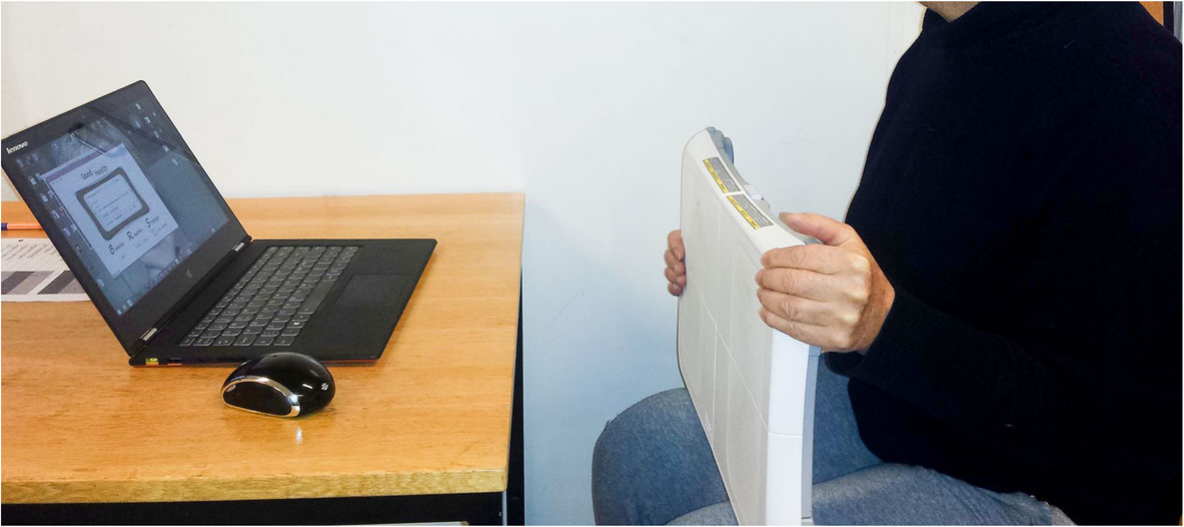
Participant seated and squeezing the upper left corner of the Nintendo Wii Balance Board

**Fig. 2:**
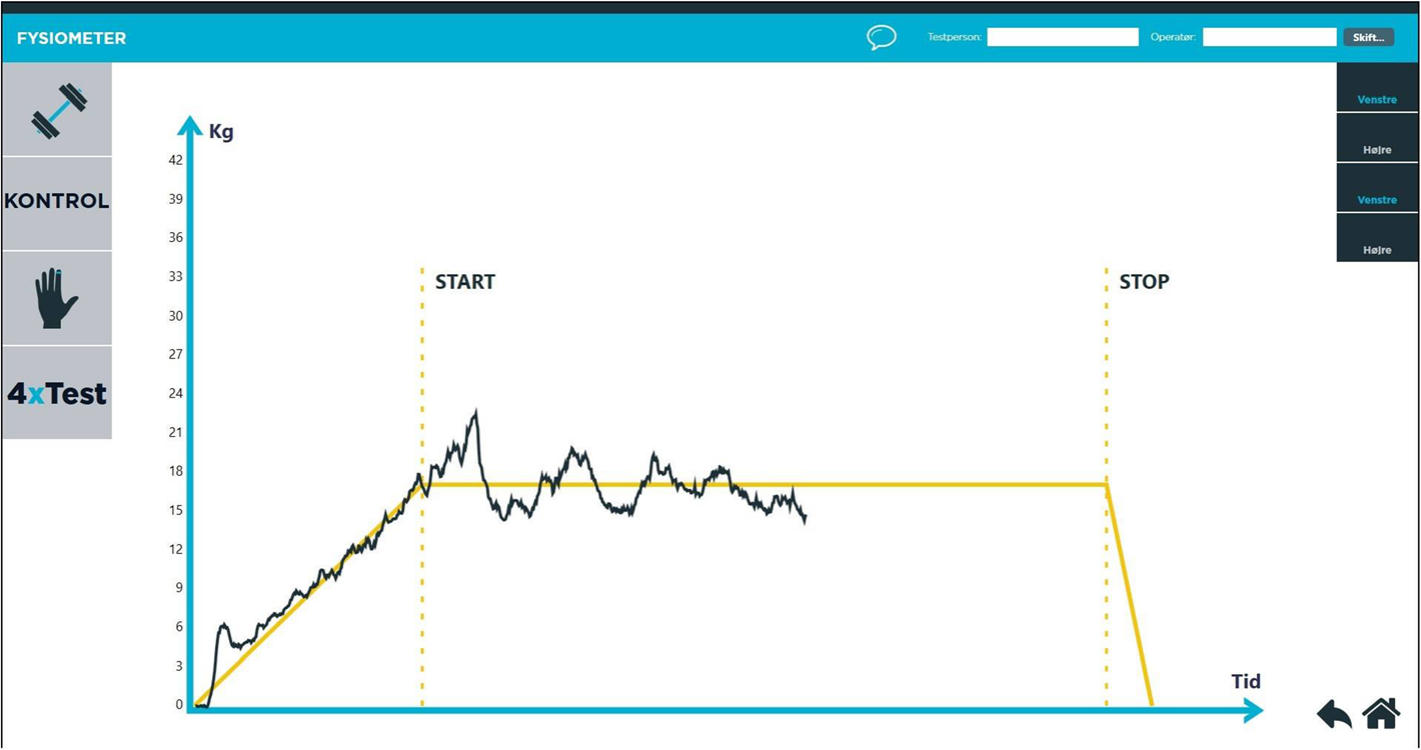
Screenshot showing the force-time curve (black) and the target force line (yellow) in the FysioMeter software

Prior to testing we measured weight and collected information on height, handedness, smoking history and number of prescribed drugs taken daily. All test procedures were performed independent of each other during home visits in the old participants and on the Oslo University campus for the young participants. The raters (Andreas Wahl Blomkvist (AWB) and Fredrik Eika (FE), medical doctors) were involved in all tests performed. Initially, maximum isometric handgrip strength was measured for each hand in order to determine MVC using a method previously described and validated [36]. The participants were seated on a standard chair and held the WBB on their lap (Fig. 1). The order of handgrip steadiness measurements at 5%, 10% or 25% of MVC as target force was randomized to avoid order effects. Steadiness at low percentages of MVC (e.g. 5% and 10%) was chosen because it has been shown to vary the most with age and gender [15, 41]. 25% of MVC was chosen because Lodha et al. [5] recommended this measure as most objective assessment of steadiness when making comparisons between chronic stroke patients and age-matched controls. The software visualized a horizontal yellow target line (20 s in duration) corresponding to the target force in a force-time curve window. An ascending line of five seconds preceded this yellow target line, which ended with a five seconds descending line as shown in Fig. 2. Participants were told to squeeze the corner of the WBB with sufficient force such that their visual force curve, produced in real-time (black graph in Fig. 2), was superimposed on the yellow target line. Furthermore, the participants were instructed to focus on the task and to avoid speaking during the test. Starting with the left hand and alternating between hands, six measurements per target force were recorded in total (three for each side), giving 18 measurements per person per session. The five seconds long intro and outro, as well as the three initial seconds of the flat yellow line, were omitted from analysis to give the participant time to adjust to the flat yellow line. In total, three times 17 s of data were used for each hand and each target percentage (i.e. 5%, 10% and 25% of MVC).

For the old participants, a retest session was performed one week later with a same time-of-day approach using MVC values from the first session. Following randomization of starting target force, handgrip steadiness was tested as described above.

### Statistics

Statistical analyses were performed using the Statistical Package for the Social Sciences version 22 (SPSS Inc., Chicago, Illinois). Each side was analysed separately using the three times 17 s of data for each session. From these data, CV was calculated in two ways [1]: SD divided by the mean force (CVM) and [2] SD divided by the target force (CVT), i.e. the actual value of the 5%, 10% or 25% of MVC. CVM and CVT were multiplied by 100 to convert into percentages. Furthermore, FysioMeter estimated the area between the target force line and the force produced. This was done by calculating the approximated area between target force line (yellow) and the force-time line (black) for ~10 ms increments of time. Thus, for each side we derived three variables (i.e. CVM, CVT and Area) for each percentage of MCV (5%, 10% and 25%) for each session. Every variable and their between-session difference were tested for normality visually (histogram) and statistically (Shapiro-Wilk test). Two-sample *t* test was used to test for differences between the old and young participants. Next, paired *t* test was used to explore systematic bias between test and retest sessions. If the normality assumption was violated, Wilcoxon signed-rank test was used.

The difference between individual results and the mean result from both sessions was plotted in a simple scatter plot to check for heteroscedasticity prior to further analysis. Reliability was assessed by calculating intra-class correlation coefficient (ICC) with 95% confidence interval using absolute agreement in a two-way mixed model and the results of a single measurement. The results were interpreted on the recommended scale of poor (< 0.69), fair (0.70–0.79), good (0.80–0.89) and high (0.90–1.00) [42] reliability. Agreement was assessed by calculating the standard error of measurement (SEM), limits of agreement (LOA) and smallest real difference (SRD). SEM and LOA were calculated by multiplying the SD of the difference between each test session with $\sqrt{1-\mathrm{ICC}}$ and 1.96, respectively. SRD was calculated by multiplying the SEM value with 1.96 • √2. Lastly, the agreement measures are given in percentages by dividing them with the mean value of both test sessions multiplied by 100.

## Results

We recruited 30 old and 10 young adults. Characteristics of the two groups are presented in Table 1. All participants reported right-sided handedness. Young adults were on average stronger than older adults (Table 1). The simple scatter plots revealed no obvious signs of heteroscedasticity. The mean results for each session, results of the *t*-test and the reliability and agreement measures are given in Tables 2 and 3 for the left and right hand, respectively. The right side generally performed better for CVT and CVM than the left side (Tables 2 and 3).

**Table 1:** Group characteristics

Characteristic	Old	Young
Number (sex)	30 (63% female; 37% male)	10 (50% female; 50% male)
Age (years)	67±8	24±3
Height (cm)	171±7	174±8
Weight (kg)	80±21	72±7
BMI (kg/cm^2^)	27	24
Left Mean MVC (kg)	20.7±6	25.6±7
Right Mean MVC (kg)	22.4±6	26.7±8

BMI and MVC is body mass index and maximal voluntary contraction, respectively

**Table 2:** Results from reproducibility analysis of measures on handgrip steadiness in old adults for the left side

Measure % of MVC	Session 1	Session 2	Difference	ICC [95% CI]	SEM (SEM, %)	LOA (LOA, %)	SRD (SRD, %)
CVM 5%	8.64	8.21	0.43 (*p* = 0.007)^a^	.911 [.785–.960]	0.29 (3.4)	1.89 (22.4)	0.80 (9.5)
CVM 10%	5.03	4.56	0.47 (*p* = 0.001)^b^	.820 [.526–.924]	0.31 (6.4)	1.43 (29.7)	0.86 (17.9)
CVM 25%	2.95	2.59	0.36 (*p* = 0.003)^b^	.767 [.439–.898]	0.31 (11.3)	1.25 (46.1)	0.86 (31.8)
CVT 5%	8.79	8.56	0.23 (*p* = 0.077)^b^	.911 [.817–.957]	0.37 (4.2)	2.41 (27.7)	1.03 (11.8)
CVT 10%	5.10	4.63	0.47 (*p* = 0.004)^b^	.806 [.559–.912]	0.38 (7.7)	1.69 (34.7)	1.05 (21.6)
CVT 25%	2.89	2.54	0.35 (p = 0.003)^a^	.774 [.446–.901]	0.28 (10.2)	1.15 (42.4)	0.78 (28.5)
Area 5%	1.85	1.59	0.26 (*p* = 0.063)^a^	.604 [.300–.793]	0.44 (25.5)	1.37 (79.6)	1.22 (70.9)
Area 10%	2.09	1.72	0.37 (*p* = 0.001)^b^	.512 [.120–.751]	0.38 (20.1)	1.08 (56.5)	1,05 (55.2)
Area 25%	2.88	2.38	0.50 (*p* = 0.004)^b^	.496 [.145–.730]	0.63 (23.9)	1.74 (66.0)	1.75 (66.3)

Session 1 and Session 2 are the mean values. Difference are the difference in the mean value from session 1 to 2. ICC, SEM, LOA and SRD are intraclass correlation coefficient, standard error of measurement, limits of agreement and smallest real difference, respectively. Absolute reproducibility results are also given in percentages (e.g. SEM%) by dividing it with the grand mean for both sessions. ^a^indicates the use of Wilcoxon signed-rank test. ^b^indicates the use of paired t-test

**Table 3:** Results from reproducibility analysis of measures on handgrip steadiness in old adults for the right side

Measure % of MVC	Session 1	Session 2	Difference	ICC [95% CI]	SEM (SEM, %)	LOA (LOA, %)	SRD (SRD, %)
CVM 5%	7.93	7.54	0.39 (*p* = 0.11)^b^	.842 [.669–.925]	0.52 (6.7)	2.57 (33.2)	1.44 (18.6)
CVM 10%	4.41	4.16	0.25 (*p* = 0.15)^b^	.783 [.595–.890]	0.41 (9.5)	1.72 (40.0)	1.14 (26.5)
CVM 25%	2.52	2.37	0.15 (*p* = 0.034)^b^	.768 [.553–.885]	0.17 (7.3)	0.72 (29.6)	4.85 (19.3)
CVT 5%	7.99	7.64	0.35 (*p* = 0.041)^b^	.806 [.619–.904]	0.55 (7.0)	2.45 (31.3)	1.52 (19.5)
CVT 10%	4.51	4.22	0.29 (*p* = 0.13)^b^	.771 [.576–.884]	0.43 (9.8)	1.76 (40.3)	1.19 (27.2)
CVT 25%	2.49	2.33	0.16 (*p* = 0.012)^a^	.769 [.546–.886]	0.17 (7.1)	0.70 (29.0)	0.47 (19.5)
Area 5%	1.92	1.71	0.21 (*p* = 0.28)^a^	.431 [.098–.679]	0.62 (34.1)	1.62 (89.2)	1.72 (94.7)
Area 10%	2.11	1.79	0.32 (*p* = 0.16)^a^	.312 [−.023–.592]	0.78 (39.9)	1.84 (94.3)	2.16 (111.0)
Area 25%	2.60	2.42	0.18 (*p* = 0.27)^b^	.431 [.095–.680]	0.63 (25.2)	1.65 (65.7)	1.75 (69.7)

Session 1 and Session 2 are the mean values. Difference are the difference in the mean value from session 1 to 2. ICC, SEM, LOA and SRD are intraclass correlation coefficient, standard error of measurement, limits of agreement and smallest real difference, respectively. Absolute reproducibility results are also given in percentages (e.g. SEM%) by dividing it with the grand mean for both sessions. ^a^indicates the use of Wilcoxon signed-rank test. ^b^indicates the use of paired t-test

**Fig. 3:**
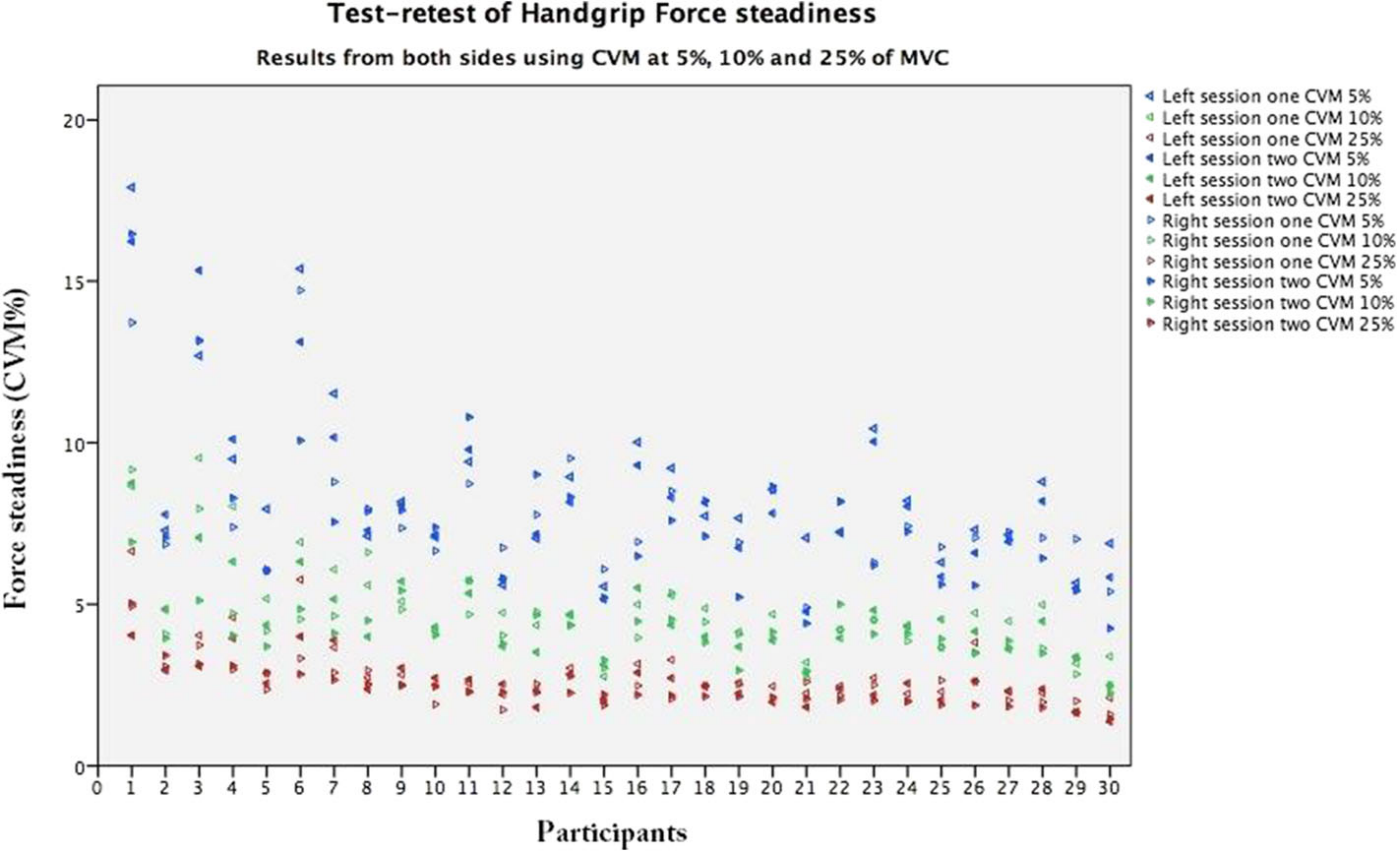
The raw test and retest data at 5 (blue), 10 (green) and 25% (red) of maximal voluntary contraction (MVC) using the coefficient of variation calculated using mean force (CVM) as an example, for both dominant (right) and non-dominant (left) sides

In Fig. 3, the raw test and retest data at 5, 10 and 25% of MVC using the CVM measure for both dominant (right) and non-dominant (left) sides are shown. There is a clear-cut trend of higher (i.e. poorer) handgrip steadiness for lower percentage of MVC as illustrated by three distinct bands of colour with the 5% (blue) at top, 10% (green) in the centre and 25% (red) at the bottom. The limited overlap even between individuals emphasises the strength of this trend. Figure 3 also shows that between-subject variation is higher with lower percentage of MVC as is illustrated by differences within colours.

An improvement from session one to session two was observed for every measure of steadiness. The improvement was statistically significant for CVM 25%, CVT 5% and CVT 25% for the right side (Table 3). For the left side, the improvement was statistically significant for every measure except CVT 5% and Area 5% (Table 2). Regarding reliability, the combined ICC for CVM, CVT and Area was 0.815, 0.806 and 0.464, respectively. Similarly, the combined ICC for all measures was 0.751, 0.667 and 0.668 for 5%, 10% and 25% of MVC, respectively. Regarding agreement, the Area measure had disproportionally high SEM, LOA and SRD results compared to CVT and CVM. The agreement for CVT and CVM was higher at 5% of MVC, except for LOA on the right side, compared with 10% and 25% of MVC.

The young adults outperformed the old adults for all measures but Area 25% MVC on the right side even though the differences did not reach statistical significance for all measures (Table 4).

**Table 4:** Age-related differences in handgrip steadiness across different measures

Measure % of MVC	Left		Difference	Right		Difference
	Old	Young		Old	Young	
CVM 5%	8.64	6.46	2.18 (*p* = .018*)	7.93	6.45	1.48 (*p* = .062)
CVM 10%	5.03	3.89	1.14 (*p* = .033*)	4.41	3.55	0.86 (*p* = .058)
CVM 25%	2.95	2.29	0.66 (*p* = .009*)	2.52	2.24	0.28 (*p* = .083)
CVT 5%	8.79	6.50	2.29 (*p* = .012*)	7.99	6.53	1.46 (*p* = .043*)
CVT 10%	5.10	3.87	1.23 (p = .031*)	4.51	3.53	0.98 (p = .043*)
CVT 25%	2.89	2.25	0.64 (p = .012*)	2.49	2.20	0.29 (*p* = .077)
Area 5%	1.85	1.16	0.69 (*p* < .001*)	1.92	1.30	0.62 (*p* = .017*)
Area 10%	2.09	1.52	0.57 (*p* = .015*)	2.11	1.52	0.59 (*p* = .031*)
Area 25%	2.88	2.57	0.31 (*p* = .450)	2.60	2.64	−0.04 (*p* = .866)

*Statistically significant at the .05 level. CVM and CVT is the calculated coefficient of variation using the mean value and the target force, respectively

## Discussion

We explored the reproducibility of handgrip steadiness measures using the WBB and compared handgrip steadiness between young and old adults. Our main findings were [1] higher reliability with 5% of MVC compared to 10% or 25% of MVC [2], higher reliability and agreement with CVM and CVT measures as compared to the Area measure [3], an improvement in handgrip steadiness between sessions [4], better handgrip steadiness among younger adults as compared to older adults. Furthermore, we found side-dependent differences in steadiness improvement, reliability, agreement and age-differences.

This is the first report to explore the reproducibility of handgrip steadiness. Previous studies used different muscle groups, most used CVM, and included fewer participants [22–27]. An improvement in force steadiness at retest was seen with most measures and statistically significant in our study. Previous studies found numerical differences that rarely reached statistical significance [22, 23] except for one [27].

We found ICCs comparable to previous studies when matching for percentages of MVC [22–24, 26] and even better when comparing with higher percentage of MVC [27]. The improved reliability for lower percentages of MVC is consistent with previous studies [24, 26] and seen across studies using similar populations and techniques [26, 27].

Also in keeping with other studies, the age-related differences in handgrip steadiness was larger for lower force levels [5, 15, 43, 44]. This range also had the highest reproducibility and thus a higher sensitivity to detect differences. The similarities to our findings are interesting even though different muscle groups are not directly comparable as they have different variability in force output (i.e. force steadiness) [19, 45]. It should be noted that some studies have found small sex differences in force steadiness [46, 47] with males being more steady than females. Thus, the small difference in sex distribution between age groups in our data may have augmented the age-difference (Table 1). Our sample size was too small to provide an estimate of the age-difference for males and females separately according to a post hoc analysis.

As expected, CVM and CVT showed parallel reproducibility. Still, we included the CVT measures as CVM is calculated independently of the target force, and it seems odd to use CVM as a test of force variability on a given target force. In fact, an excellent score using CVM may be seen while completely failing the desired target force. In addition, the CVT and CVM measures may both be relevant for future investigation as motor impairments measured by target force dependent measures (i.e. CVT) differ from the target force independent measures (i.e. CVM) [48]. The reliability and agreement of the Area measure using the WBB was poor and thus found not to be a recommendable measure.

The ICCs reflect the degree to which individuals maintain their ranked position in a sample with repeated measurements [49]. A high ICC indicates that the method is applicable for comparisons among groups of people. Our results indicate that CVM and CVT measure at 5% of MVC is useful for comparing handgrip steadiness between groups. We also found good relative reproducibility for CVM and CVT at 10% of MVC despite an increasing steadiness between sessions. This means that improvements, especially in CVM and CVT at 5% of MVC, were similar and participants did not greatly change their ranked position in the sample. It is important for future studies to investigate and/or account for this systematic improvement.

The agreement measures (SEM, LOA and SRD) addresses the degree to which repeated measurements vary in an individual. These are clinically relevant measures that indicate if it is a “true” change or if it may be due to random variation [38]. However, these measures should be interpreted in context. For instance, CVM and CVT at 5% of the SEM, LOA and SRD are on average 5%, 29% and 15%, respectively. This means that these measures will vary around 5% (SEM) of a subjects “true” value, and that the magnitude of change that may be due to random error lies within 29% (LOA) with 95% probability. Beckerman et al. [50] introduced the SRD to indicate whether a subsequent measurement represents a real change and not random variation. SRD represents the smallest change in measurement, which can be attributed to a real change in the parameter assessed. A SRD of 15% means that the subsequent force steadiness measurement must exceed a 15% difference to indicate a true change in force steadiness. Also, we found a mean difference between sessions that was more prominent for the left than for the right. The side-dependent difference might be due to a training effect. In keeping with the poorer side being more sensitive to a training effect, the right side outperformed the left side on CVT and CVM.

The SEM, LOA and SRD for CVM at 5% of MVC were 3.4/6.7%, 22/33% and 9.5/19% respectively for the left/right sides. The cause for this difference cannot be deduced from our results. One speculation is that by testing the left side first on each trial for a given target force, the participant needed some time to adjust for the new target line and, thus, the left side avoided an immediate learning effect to a larger degree than the right. Another possible reason is that the right side, as it is the preferred hand, will in all likelihood show less between-subject variation and, hence, lower reliability. These explanations are not mutually exclusive, but can easily be explored in other experimental set ups. A final speculation on noticeable side-difference can be related to the target force most frequently used in everyday life that is approximately 25% of MVC [51]. Thus, this measure has the lower within-subject variation and the lowest training effect, which could explain the higher agreement for CVM and CVT at 25% of MVC on the right side. In fact, opposite to the left side, the agreement measure at the right side is noticeably better for 25% of MVC than for 10% of MVC.

Our study and method have limitations. Using the WBB does not allow adjustments for differences in hand size, which also may have implications for sex differences in steadiness. Second, the method relies on visuomotor feedback. Some of the age-related force steadiness differences in hand muscles may be attributable to processing of visuomotor information [52]. Specifically, old adults, as opposed to young adults, seem to have significantly lower steadiness with visual feedback compared to no visual feedback at lower forces [52]. Still, the clinical relevance may be independent of the cause of the age-related decrease in force steadiness. Third, the observed systematic improvement and its side-dependent difference, as well as the influence of the starting hand, are all aspects which require further investigation. Different experimental protocols can easily be developed to understand and likely adjust for these factors.

The study also has strengths. The WBB is a relatively cheap, widely available and portable device, and the *FysioMeter* software is accessible to support the method. Using this method, we reported multiple measures across different target forces, and we separate true effects from random results as the study included a sufficient number of participants to conduct reliability and agreement statistics [40].

## Conclusion

This reproducibility study on handgrip force steadiness reported good reliability and acceptable agreement for CVM and CVT measures at 5% of MVC in older adults. We found side-dependent differences in force steadiness improvement, reliability, agreement and age-differences, and we detected a systematic difference in the mean with sessions and with age. With these caveats in mind, we conclude that the WBB method is a reliable instrument for measuring handgrip steadiness using CVM and CVT at low MVC. More research is needed to understand and characterize how handgrip steadiness is related to age, handedness and over repeated assessments.

## Abbreviations

CVM: Coefficients of variation calculated from the mean force produced
CVT: Coefficients of variation calculated from the target force
ICC: Intraclass correlation coefficient
LOA: Limits of agreement
MVC: Maximum voluntary contraction
SEM: Standard error of measurement
SRD: Smallest real difference)
WBB: Wii Balance Board

## Footnotes

1 ApEn is a regularity statistic that gives a measure of the time-dependent structure of the force signal
